# Slot Allocation Protocol for UAV Swarm Ad Hoc Networks: A Distributed Coalition Formation Game Approach

**DOI:** 10.3390/e27030256

**Published:** 2025-02-28

**Authors:** Liubin Song, Daoxing Guo

**Affiliations:** 1 College of Communications Engineering, Army Engineering University of PLA, No. 2 Biaoying, Ruijin Road, Qinhuai District, Nanjing 210007, China; waterslb@163.com; 2 Institute of Microelectronics, Chinese Academy of Sciences, No. 3 Beitucheng West Road, Beijing 100029, China

**Keywords:** UAV swarm ad hoc network, slot allocation, coalition formation game

## Abstract

With the rapid development of unmanned aerial vehicle (UAV) manufacturing technology, large-scale UAV swarm ad hoc networks are becoming widely used in military and civilian spheres. UAV swarms equipped with ad hoc networks and satellite networks are being developed for 6G heterogeneous networks, especially in offshore and remote areas. A key operational aspect in large-scale UAV swarm networks is slot allocation for large capacity and a low probability of conflict. Traditional methods typically form coalitions among UAVs that are in close spatial proximity to reduce internal network interference, thereby achieving greater throughput. However, significant internal interference still persists. Given that UAV networks are required to transmit a substantial amount of safety-related control information, any packet loss due to internal interference can easily pose potential risks. In this paper, we propose a distributed time coalition formation game algorithm that ensures the absence of internal interference and collisions while sharing time slot resources, thereby enhancing the network’s throughput performance. Instead of forming a coalition from UAVs within a contiguous block area as used in prior studies, UAV nodes with no interference from each other form a coalition that can be called a time coalition. UAVs belonging to one coalition share their transmitting slots with each other, and thus, every UAV node achieves the whole transmitting slots of coalition members. They can transmit data packets simultaneously with no interference. In addition, a distributed coalition formation game-based TDMA (DCFG-TDMA) protocol based on the distributed time coalition formation algorithm is designed for UAV swarm ad hoc networks. Our simulation results verify that the proposed algorithm can significantly improve the UAV throughput compared with that of the conventional TDMA protocol.

## 1. Introduction

Sixth-generation (6G) wireless networks are expected to provide large-scale connectivity for millions of interconnected devices, providing different quality-of-service (QoS) requirements, ubiquitous coverage, high embedded artificial intelligence (AI), efficient energy utilization, and adaptive network security [1]. Advances in manufacturing technology have led to a reduction in the cost of individual unmanned aerial vehicles (UAVs) while enhancing their mobility, deployment efficiency, and flexibility [2,3]. UAVs should play an important role in the 6G ecosystem, as flight equipment is expected to occupy dense airspace for transportation, monitoring, network relay, and other tasks [4]. UAVs communicate with other UAVs and satellite stations, forming a space–air network, which can become a part of 6G heterogeneous networks. The deployment of large-scale UAVs integrated with highly reliable networks and advanced intelligent collaboration algorithms has become a prominent research focus in recent years. A UAV swarm composed of large-scale small and low-cost drones is expected to achieve higher and greater performance relative to independent drones through collaboration and information exchange.

In many applications, such as surveillance, reconnaissance, and search and rescue, UAV swarms need to constantly exchange data with each other and with rear command posts during the execution of tasks, to improve task performance, survivability, and scalability. Especially in intensive, dynamic, and time-critical tasks, the need for wide bandwidth, high capacity, high date rates, and low latency is increasing [5]. A distributed network structure is more suitable for large-scale UAV swarm networks. This is because the distributed network has better anti-destruction and self-healing capabilities, and does not stop working due to the destruction of a single node [6]. A heterogeneous network includes a UAV swarm ad hoc network, and the satellite network is a typical application method in offshore and remote areas. However, UAV swarms have many challenges when resource management is implemented in such networks. First, the UAV nodes of the swarm are densely distributed, and interference among the UAV swarm seriously affects the performance of the ad hoc network. Second, the network topology may change rapidly and dynamically due to the high mobility of the drones, resulting in frequent switching and mutual interference of the network connections. Third, in addition to mutual interference within the UAV swarm that may cause a complex electromagnetic environment, the existence of malicious interference also causes complications.

A UAV swarm requires the constant interaction of location and status information for swarm control algorithms such as path planning and collision avoidance. These algorithms require high frequency and accuracy of messages. Collision and packet loss may significantly reduce the coordination algorithm performance of UAV swarms. Thus, addressing the internal interference of UAVs in a dynamic-topology environment has become the core key problem of large-scale UAV swarm ad hoc networks.

UAVs are increasingly being used in communication networks, with typical applications such as IoT data acquisition and 6G flying base stations. In these applications, UAV path planning is an important topic. The communication supported by unmanned aerial vehicles (UAVs) has become a highly attractive technology in the field of wireless sensor networks (WSNs) for efficient data collection [7,8,9]. Collecting data from ground-distributed sensor network is one of the key technologies of WSNs. The typical goals of trajectory optimization are to minimize the task time of unmanned aerial vehicles [10,11], minimize energy consumption during flight [12,13], maximize the amount of data collected [14], and maximize the number of service sensors [15]. UAV-assisted 5G/6G networks can improve network efficiency and flexibility by providing seamless on-demand ultra-high-capacity connections for elastic traffic in these areas. This is crucial because trajectory planning determines the overall travel time, the services provided to the GU, and the energy consumed by the drone [16]. The optimization problem of drone trajectory planning is similar to the well-known Traveling Salesman Problem (TSP) [15,16,17] and Vehicle Routing Problem (VRP) [18].

A simple method that ensures collision-free transmission and guarantees communication latency between UAVs is to use the time division multiple access (TDMA) protocol. The fixed TDMA protocol can avoid conflicts, but the time slot cannot be flexibly scheduled, which is inefficient in large-scale UAV swarm networks. To solve this problem, some TDMA-based protocols for vehicle ad hoc networks have been proposed [19,20,21,22]. These protocols eliminated many collisions by dividing different driving direction nodes into different time slot sets. However, these protocols of slot partitioning in VANETs were not suited for UAV swarm networks, because the movement direction of UAVs was arbitrary. In [23], a collision-free MAC protocol, termed CF-MAC, was introduced. This protocol enabled UAVs equipped with half-duplex radios and omnidirectional antennas to swiftly access networks while employing a region-marking scheme that diminished the collision probability to nearly zero. In [24], a token-based protocol was proposed, which used full-duplex radios and multipacket reception capability to achieve a higher throughput and a lower delay. A concurrent transmission MAC protocol that works by combining millimeter, directional antenna and TDMA for UAV ad hoc networks was proposed to meet the requirements of UAV ad hoc networks in terms of bandwidth, data rate and delay [25]. These protocols compromised on either conflict or flexible scheduling, or needed the addition of full-duplex, directional antennas, millimeter waves, or even shared location information, and were not widely applied due to their complexity.

Game theory is a branch of applied mathematics that is a natural and powerful tool for analyzing the interactions between multiple decision-makers. It is mainly used to study competition or cooperative behavior in economics [26]. The resource management problem for large-scale UAV communication networks, from a game-theoretic perspective, was investigated in [6], and it was exactly aligned with the distributed and autonomous nature of such networks. A network-side self-organized coalition formation approach for intra-tier interference mitigation, primarily through neighborhood cooperation, was proposed in [27]; by forming disjoint coalitions, small-cell access points (SAPs) were able to mitigate the interference within a coalition and thus improved their achievable data rates. A distributed coalition formation algorithm for cooperation in ad hoc networks was proposed in [28], which used the joining and leaving rules for the choice of each node to assure that the newly formed coalitional structure could converge to a stable structure. The coalition formation game proposed in the above study could coordinate internal interference in the network by aggregating adjacent nodes to form an independent coalition, but they reduced only the degree of internal interference and could not avoid interference among nodes. To solve the challenges of resource management and routing protocols, a cross-layer optimization method was presented with a novel mean field game (MFG) in [29], which could select the optimal data transmission path through the management and allocation of frequency resources and power resources. The algorithm of forming coalitions required full channel status information and the results of each iteration, which was difficult to obtain in ad hoc networks. Ref. [27] obtained CSIs and iteration results through a central base station, while [28] did not explain where these were obtained. These methods typically form coalitions among UAVs that are in close spatial proximity to reduce internal network interference, thereby achieving greater throughput. However, significant internal interference still persists.

In our opinion, the UAV swarm network is a specific distributed communication system, and large amounts of distributed coordination signals and control data for drones must be transmitted in the network. The UAV swarm network must be a reliable and stable system for special applications such as rescuing, searching, attacking, etc. Packet loss, especially the collision loss of coordination signals, may cause networks to become unstable, which is especially important for distributed networks because the stability and reliability of distributed networks are inherently difficult. Hence, the premise of designing a stable and reliable UAV swarm ad hoc network is the absence of conflict and collision.

In this paper, a distributed slot allocation algorithm for UAV swarm ad hoc networks based on time coalition formation game is proposed. Instead of forming coalitions from UAVs within a contiguous block area as in prior studies, UAV nodes with no interference from each other form a coalition, which can be called a time coalition. UAVs belonging to one coalition can share their transmitting slots with each other, and thus, every UAV node in the coalition achieves the whole transmitting slots of the coalition members. They can transmit data packets simultaneously with no interference. By dividing the UAV nodes into different time coalitions, interference between UAV nodes can be avoided, and thus, the performance of network throughput and time delay is improved. The main contributions of our paper are summarized as follows:

(1) We formulate the slot allocation problem of the UAV swarm ad hoc network based on the TDMA protocol as a coalition formation game among UAV nodes with different transmission time slots. Within the framework of the proposed game, UAV nodes with no interference from each other form a coalition, share their time slots, and transmit data packets simultaneously.
(2) A simple distributed coalition formation algorithm based on the join-and-leave rule is proposed, through which each UAV node autonomously determines the most beneficial coalition to join. Subsequently, the whole network quickly converges to a final stable partition.
(3) Based on the distributed time coalition formation algorithm, we design a TDMA MAC protocol (called DCFG-TDMA) for the UAV swarm ad hoc network. UAV nodes with a DCFG-TDMA protocol can achieve time slots adaptively with dynamically changing network topology.

The rest of the paper is arranged as follows. Section 2 provides a comprehensive review of the existing literature pertinent to the topic of this paper. In Section 3, we present the system model and problem formulation. In Section 4, we describe the proposed coalition formation game model and the distributed coalition formation algorithm. In Section 5, the TDMA protocol based on distributed formation game for UAV swarm ad hoc networks is proposed. The simulation configurations and numerical results are discussed in Section 6. Finally, our conclusions are drawn in Section 7. For convenience, the abbreviations used in this paper are listed in Table 1.

## 2. Related Works

Recently, numerous methods have been proposed to address the issue of internal interference among nodes in UAV networks. Broadly speaking, these can be categorized into the following types.

### 2.1. MAC Based on CSMA Mechanism

Carrier-Sense Multiple Access with Collision Avoidance (CSMA/CA) stands as one of the foundational medium access control (MAC) protocols for ad hoc networks. Its CSMA-based variants have been widely adopted in unmanned aerial vehicle (UAV) networks due to their adaptability and efficiency. Nonetheless, as the network scales and the number of nodes increases, the likelihood of collisions escalates, posing significant challenges to maintaining optimal performance. In pursuit of maximizing network throughput, ref. [30] introduces an optimized distributed CSMA/CA strategy, enhanced by opportunistic Reconfigurable Intelligent Surface (RIS) assistance, grounded in statistical optimization principles. Addressing the issue of collision probability, ref. [31] proposes a Non-Orthogonal Multiple Access (NOMA)-based CSMA/CA (NCSMA) protocol. This innovative NCSMA protocol leverages Zadoff–Chu (ZC) sequences alongside a NOMA Power Coefficient Allocation (NPCA) frame to meticulously select NOMA nodes and allocate power levels, thereby circumventing packet collisions typically induced by random selection processes. The NCSMA protocol demonstrates a marked improvement in normalized saturation throughput and a significant reduction in average packet delay.

Despite these advancements, the integration of either RIS assistance or NOMA technology into existing UAV systems remains a complex endeavor, necessitating a substantial developmental timeline under current technological and operational constraints.

### 2.2. MAC Based on TDMA Mechanism

Building upon the foundational design of the ADHOC MAC protocol [19], Omar et al. introduced VeMAC [20], a TDMA-based MAC protocol tailored specifically for Vehicular Ad Hoc Networks (VANETs). Capitalizing on the characteristic stability of vehicles traveling on highways and the consistent network topology in the same direction, VeMAC ingeniously partitions time slots into three distinct segments. Two of these segments are allocated for vehicles moving in opposite directions, while the remaining segment is reserved for roadside units. Each node accesses a different set of slots based on its identity and direction, thereby significantly reducing the potential for collisions. In a similar vein, to address the stringent bandwidth, data rate, and delay requirements of UAV ad hoc networks, a concurrent transmission MAC protocol was proposed in [25], which integrates millimeter-wave technology, directional antennas, and TDMA. A unifying slot assignment protocol (USAP) was proposed to manage the TDMA slot and channel assignments for optimal operation in the face of changing topologies and traffic requirements [32]. Ref. [33] proposed a multi-agent DRL-enhanced USAP method to improve network utility in distributed implementation. Ref. [34] introduced a dual-cluster-head-based medium access control (DCHMAC) scheme for large-scale UAV networks, demonstrating significant throughput improvement. The inter-cluster communication and the one-hop communication uses a CSMA scheme. The nodes whose distances were beyond the one-hop range adopted TDMA.

These protocols have successfully mitigated numerous collisions by segregating nodes based on their driving directions into separate time slot sets. However, the slot-partitioning strategies effective in VANETs are not directly transferable to UAV swarm networks. The inherent arbitrariness of UAV movement directions means that even with the VeMAC method, UAV networks still face collision issues. Furthermore, while the approach outlined in [25] demonstrates superior performance, its practical application in UAV systems is hindered by the complexities associated with millimeter waves and directional antennas.

### 2.3. Method Based on Game Theory

The resource management challenges in large-scale UAV communication networks were explored from a game-theoretic perspective in [6], aligning perfectly with the distributed and autonomous nature of such networks. In [27], a network-side self-organized coalition formation approach was introduced to mitigate intra-tier interference primarily through neighborhood cooperation. By forming disjoint coalitions, small-cell access points (SAPs) were able to reduce interference within each coalition, thereby enhancing their achievable data rates. Similarly, ref. [28] proposed a distributed coalition formation algorithm for cooperation in ad hoc networks, utilizing joining and leaving rules to ensure that each node’s choice led to a stable coalitional structure.

While the coalition formation games proposed in these studies effectively coordinated internal interference by aggregating adjacent nodes into independent coalitions, they only managed to reduce the degree of internal interference rather than eliminating it entirely. From our perspective, given that UAV networks are tasked with transmitting a significant volume of safety-critical control information, any data loss due to internal interference could result in substantial and potentially catastrophic consequences. Therefore, the ultimate design goal for UAV swarm networks should be the establishment of a completely collision-free and highly reliable network.

### 2.4. Method Based on Reinforcement Learning

Reinforcement Learning (RL), as an effective method for time-varying dynamic systems, is gradually being applied to resource allocation in wireless networks. Ref. [35] studied the throughput and fairness issues in multi-channel centralized random access systems and proposed a distributed multi-channel access protocol based on multi-agent reinforcement learning (RL) to enhance throughput and fairness among active users. Addressing the high-concurrent-access problem in industrial wireless networks that meet strict real-time and reliable communication requirements, ref. [36] proposed a Deep Reinforcement Learning-based Dynamic Priority Multichannel Access (DRL-DPMCA) algorithm, which ensures the highest channel access probability and lowest queue delay for high-priority industrial equipment while minimizing access conflicts and achieving near 100% channel utilization. To tackle the problem of agents being unable to identify and explore useful information in sparse wireless environments, ref. [37] proposed a Cooperative Multi-Agent Exploration (CMAE) framework that projects the network state space into a low-dimensional space rather than learning strategies in a high-dimensional space, with nodes and BSs collaboratively exploring unexplored wireless network states to jointly learn channel access and signaling strategies, achieving good throughput performance and low collision rates.

However, the application of reinforcement learning algorithms in access control is still in the theoretical stage, and due to their issues pertaining to computational complexity, timeliness, and deployment, it is challenging to apply them to practical systems.

## 3. System Model and Problem Formulation

### 3.1. System Model

**Figure 1:**
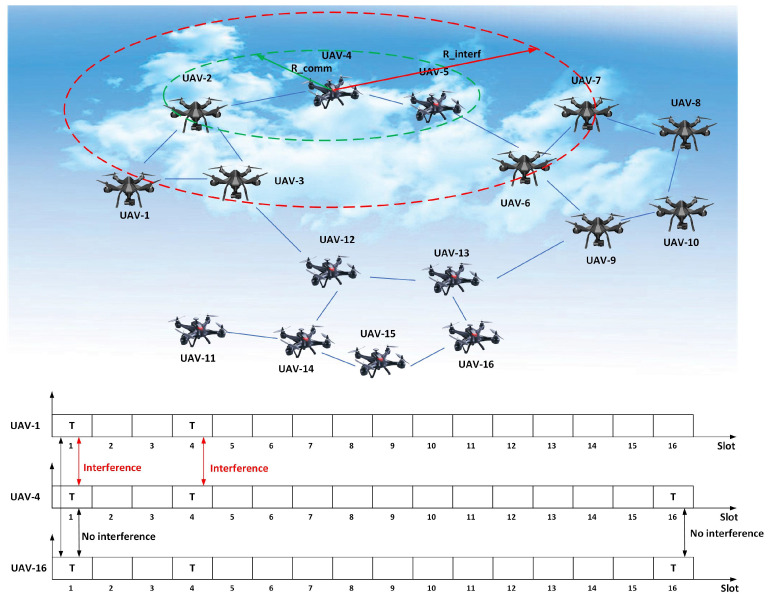
An illustration of the multi-UAV network, showing the interference of UAV communication slots.

As shown in Figure 1, a UAV swarm ad hoc network formed of a large number of drones is considered, where *S* unmanned aerial vehicles (UAVs) are randomly distributed in the task area. Let $N=\left\{1,2,\ldots ,N\right\}$ represent the index set of the UAVs in the flying ad hoc network scenario as a drone formation. In the UAV ad hoc network, there is no center node and no cluster node, and every node has equal status. Each UAV is equipped with one omnidirectional antenna. All nodes work on the same frequency point, and TDMA mechanisms are adapted in the mac protocol of all UAVs for sharing the frequency point. Each node works in the time division duplex (TDD) mode, which transmits or receives packets at one slot only. There is one channel and there are *N* slots available for the multi-UAV network. The set of available time slots is denoted by $T=\left\{1,2,\ldots ,N\right\}$.

#### 3.1.1. Channel Model

In the UAV swarm ad hoc network, the main wireless communication links are air-to-air (A2A) links. For A2A communication, the free-space propagation model is often utilized, since A2A links are mainly dominated by line-of-sight (LOS) links [3]. Thus, the channel power gain depends mainly on the distance between UAVs for simplicity. The path loss between UAV-*i* and UAV-*j* can be expressed by (1):

(1) $$\begin{array}{c} L_{ij}=\eta_{los}{\left(\frac{4\pi f}{c}\right)}^{2}d_{ij}^{2} \end{array}$$

where $\eta_{los}$ represents the attenuation factors corresponding to the LOS link, while *f* is the carrier frequency and *c* denotes the speed of light.

Given the bandwidth *B*, the maximum transmission rate between UAVs *i* and *j* can be calculated by (2):

(2) $$\begin{array}{c} C_{ij}=B\log_{2}\left(1+\frac{P_{t}}{L_{ij}\sigma^{2}B}\right) \end{array}$$

where *B* is the bandwidth of the network, $P_{t}$ is the transmit power of each UAV node, and σ is the Gaussian noise distributed with zero mean. We assume that the gain of omnidirectional antennas is one.

#### 3.1.2. Analysis of Communication and Interference

Suppose that all UAV nodes transmit data packets at a fixed rate $R_{0}$. Then, UAV-*j* can correctly receive the packets from UAV-*i* if the maximum transmission rate between UAVs *i* and *j* is $C_{ij}\geq R_{0}$:

(3) $$\begin{array}{c} C_{ij}=B\log_{2}\left(1+\frac{p_{t}}{L_{ij}\sigma^{2}B}\right)\geq R_{0} \end{array}$$

(4) $$\begin{array}{c} d_{ij}\leq \frac{c}{4\pi f\sigma }\sqrt{\frac{p_{t}}{B\left(2^{R_{0}/B}-1\right)\eta_{los}}} \end{array}$$

Thus, the UAVs within the range $d_{C}$ of UAV-*i* can communicate correctly, where $d_{C}$ is given in (5).

(5) $$\begin{array}{c} d_{C}=\frac{c}{4\pi f\sigma }\sqrt{\frac{p_{t}}{B\left(2^{R_{0}/B}-1\right)\eta_{los}}} \end{array}$$

The power of the signal of UAV-*i* after transmitting a distance *d* is given in (6).

(6) $$\begin{array}{c} p_{r}=\frac{p_{t}}{\eta_{los}{\left(\frac{4\pi f}{c}\right)}^{2}d^{2}} \end{array}$$

Then, a UAV node within the range *d* of UAV-*i* receives the interference power $p_{r}$ if it does not communicate with UAV-*i*. As an approximation, when the power of the interference signal is lower than the noise power, it has no effect on communication. Thus, the interference range $d_{I}$ of UAV-*i* is (7).

(7) $$\begin{array}{c} d_{I}=\frac{c}{4\pi f\sigma }\sqrt{\frac{p_{t}}{\eta_{los}B}} \end{array}$$

**Figure 2:**
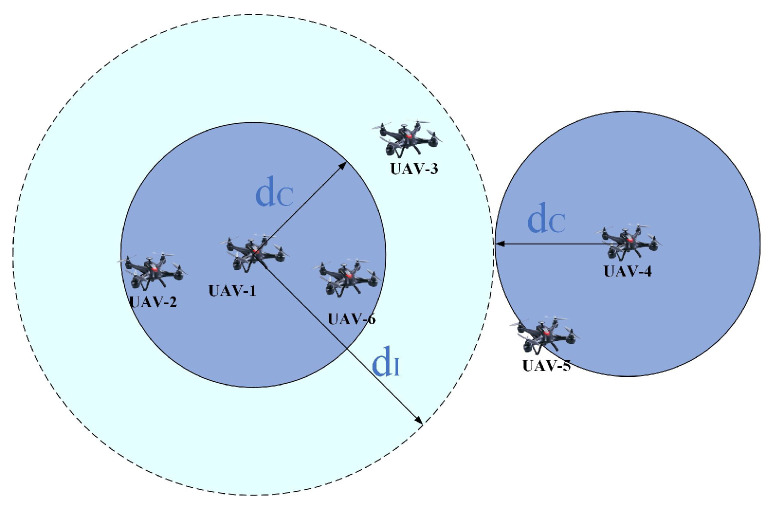
An illustration of communication distance and interference distance.

As shown in Figure 2, UAV-2 is in the communication range of UAV-1, and UAV-3 is in the interference range of UAV-1. UAV-5 is in the communication range of UAV-4, but it is outside the interference range of UAV-1. Thus, UAV-4 and UAV-1 can simultaneously transmit signals to UAV-2 and UAV-5, respectively. UAV-3 and UAV-5 cannot transmit a signal when UAV-1 is transmitting a signal because this may affect the reception of UAV-6. In other words, when the distance between two UAVs is longer than $d_{C}+d_{I}$, they can transmit signals in the same time slot.

#### 3.1.3. Network Topology

Consider the network topology which is shown in Figure 1. Sixteen UAV nodes form a flying ad hoc network. Here, the distance between two UAVs, i.e., *i* and *j*, is denoted by $d_{ij}$. If the distance $d_{ij}$ is less than the communication threshold, they can communicate with each other. A collision occurs when the distance $d_{ij}$ is less than the collision threshold $d_{C}+d_{I}$.

Thus, the communication graph is defined as $G_{C}=\left\{V,E_{C}\right\}$, where *V* represents the UAV set, $V=\left\{v_{1},v_{2},\ldots ,v_{N}\right\}$, and $E_{C}$ is the edge set, i.e., $E_{C}=\left\{\left(v_{i},v_{j}\right)|v_{i},v_{j}\in V\right\}$. Similarly, the collision graph is defined as $G_{I}=\left\{V,E_{I}\right\}$, and $E_{I}$ is the edge set, i.e., $E_{I}=\left\{\left(v_{i},v_{j}\right)|{d_{ij}\leq d_{C}+d_{I},v}_{i},v_{j}\in V,v_{i}\neq v_{j}\right\}$.

Figure 1 illustrates the interference of three nodes (UAV-1, UAV-4, and UAV-16) in every communication slot. Slot 1 is the transmitting slot belonging to UAV-1. For higher slot utilization, if UAV-4 and UAV-16 also transmit signals in slot 1, UAV-4 will interfere with UAV-1 where UAV-16 will not, and UAV-16 does not interfere with UAV-4. Similarly, if UAV-1 and UAV-4 transmit signals in slot 4, interference occurs for $d_{1,4}\leq d_{I}$.

### 3.2. Problem Formulation

The UAVs in the network adopt the Time-Division Multiple Access (TDMA) scheme to transmit packets. As shown in Figure 1, UAV-4 and UAV-2 have overlapping coverage areas, and similarly, UAV-15 and UAV-16 also have overlapping coverage areas. When UAV-4 and UAV-2 individually transmit packets at the same slot, UAV-4 causes interference with UAV-2. In contrast, when UAV-16 and UAV-1 transmit packets in the same slot, co-channel interference does not happen. Because UAV-16 is far from UAV-1, the signal of UAV-20 cannot interfere with the UAVs to which UAV-1 transmits.

Assume that we allocate $K_{n}$ slots for UAV-*n*, where $\;1\leq K_{n}\leq N$. Consider $T_{n}$ to be the corresponding slots selected by UAV-*n*, where $T_{n}=\left\{t_{1},t_{2},\ldots ,t_{K_{n}}\right\},t_{i}\in T,1\leq i\leq K_{n}$.

For an arbitrary individual UAV-*n* at an arbitrary time slot *t*, $t\in T_{n}$, we denote $R_{n}\left(t\right)$ as the throughput at time slot t of UAV-*n*, where $R_{n}\left(t\right)$ is given in (8).

(8) $$\begin{array}{c} R_{n}=\left\{\begin{array}{ccc} 0, & & \exists i\in N,{i\neq n,d}_{in}\leq d_{C}+d_{I},t\in T_{i}\\ R_{0}, & & else \end{array}\right. \end{array}$$

$R_{0}$ is the fixed transmitting rate of each UAV, and $T_{i}$ is the corresponding slots selected by UAV-*i*. We note that the time slot length is normalized to 1. For an arbitrary individual UAV-*n*, the total throughput can be calculated as

(9) $$\begin{array}{c} {Th}_{n}=\sum_{t\in T_{n}}R_{n}\left(t\right) \end{array}$$

Then, the throughput of the whole multi-UAV network is

(10) $$\begin{array}{c} Th=\sum_{n=1}^{N}\sum_{t\in T_{n}}R_{n}\left(t\right) \end{array}$$

An arbitrary individual UAV prefers to select more transmission time slots so that it can achieve higher throughput. But the higher number of transmission time slots will bring more collision at the same slot, which may cause a reduction in the throughput. From a multi-UAV network viewpoint, a higher number of selected time slots of each UAV yields more transmission opportunities. Thus, our aim is to maximize the aggregate throughput of the network, as 

(11) $$\begin{array}{c} P1:\max\sum_{n=1}^{N}\sum_{t\in T_{n}}R_{n}\left(t\right) \end{array}$$

From a time slot viewpoint, we want more UAVs to transmit at the same slot, and we do not want them to interfere with each other. Denote $N_{t}$ as the set of UAVs which select time slot *t* to transmit. For an arbitrary individual time slot *t*, the throughput of the whole network is

(12) $$\begin{array}{c} R\left(t\right)=\sum_{n\in N_{t}}R_{n}\left(t\right) \end{array}$$

Then, the throughput of the whole multi-UAV network can also be written as

(13) $$\begin{array}{c} Th=\sum_{t=1}^{N}\sum_{n\in N_{t}}R_{n}\left(t\right) \end{array}$$

Obviously, the large no-collision UAV set of time slot *t* induces high network throughput. The problem of maximizing the aggregate throughput of the network transforms to find a group of the largest no-collision UAV set at time slot *t*.

(14) $$\begin{array}{cc} \; & P2:\max\sum_{t=1}^{N}\sum_{n\in N_{t}}R_{n}\left(t\right) \end{array}$$

(15) $$\begin{array}{cc} s.\;t.\; & \;\forall i,j\in N_{t},d_{ij}>d_{C}+d_{I} \end{array}$$

P1 and P2 are equivalent. Problem 1 finds the maximum value of the sum of the throughput of all nodes, and problem 2 finds the maximum value of the sum of the rates on all time slots from the perspective of time slots.

## 4. The Coalition Formulation Game Model

As discussed in Section 2, the problem of P1 can be transformed to find a group of the largest no-collision UAV set at time slot *t*. We denote the no-collision UAV set as a UAV coalition. The UAV which joins the no-collision UAV set can achieve the transmitting time slot of the current time slot *t*. At each time slot, the UAVs have a strong incentive to cooperate and to form coalitions to improve their throughput.

Assume that in the TDMA multi-UAV network, each UAV-*i* has a fixed transmitting time slot *i*, $i\in \left\{1,2,\ldots ,N\right\}$. When a UAV coalition is formed, the member in the coalition can transmit packets on the time slot of the whole coalition member, since they have no collision with each other. Considering that all the *N* UAVs are selfish and rational, we formulate their decision-making process as a coalition formation game, unlike the canonical coalition game.

### 4.1. Coalition Formation Basic Concepts

**Definition** **1.** *A distributed coalition game $G_{D}$ with non-transferable utility (NTU) is defined by an ordered pair (S,V), where S represents the finite set of players (i.e., UAVs), and V is a partition function, $V:\prod \left(S\right)\to R^{S}$ that maps, for every partition $\prod \left(S\right)$ and coalition $Q_{K}\in \prod \left(S\right),\;Q_{K}\neq Ø$, to a closed convex subset of $R^{|Q_{K}|}$ that contains the payoff vectors that players in a coalition $Q_{K}$ can achieve.*

Here, we note that the empty set in S is 0, namely, $v\left(Ø\right)=0$. A coalition denoted by $S_{K}$ is defined as a nonempty subset of players over $S=\left\{1,2,\ldots ,N\right\}$, where $S_{K}\subseteq S$. Each player i∈S can freely join only one coalition.

In the proposed game $G_{D}=\left(S,\;V\right)$, all the *N* UAVs can autonomously organize according to the environment and choose their potential members with which to cooperate and to form coalitions. Subsequently, all the members in a coalition share their initial fixed time slot resources, that is, the UAVs in a coalition $S_{K}$ can achieve $\left|S_{K}\right|$ transmitting time slots.

**Definition** **2.** *In the disjoint coalition game $G_{D}=\left(S,\;V\right)$, a coalition structure denoted by π is defined as a set of $\pi =\left\{S_{1},S_{2},\ldots ,S_{M}\right\}$, where M is the number of coalitions, $1\leq M\leq N,{⋃}_{i=1}^{M}S_{i}=S$. Here, $\exists S_{i},S_{j}\in \pi ,i\neq j$ such that $S_{i}\cap S_{j}=Ø$.*

The grand coalition is defined as a coalition containing all UAVs. In the network, $2^{N}-1$ distinct nonempty coalitions and $D_{N}$ different coalitional structures for *N* players can be formed:

(16) $$\begin{array}{c} D_{N}=\left\{\begin{array}{ccc} \sum_{j=0}^{N-1}C_{N-1}^{j}D_{j}, & \;\; & N\geq 1\\ 1, & \;\; & N=0 \end{array}\right. \end{array}$$

**Definition** **3.** *For a given partition π, we define the utility function of the coalitional structure π as $v\left(\pi \right)=\sum_{j=1}^{M}v\left(S_{j},\pi \right)$, where $v\left(S_{j},\pi \right)$ denotes the utility function of the coalition $S_{j}\in \pi$. In such a case, the utility of coalition $S_{j}$ can be defined as $v\left(S_{j},\pi \right)=\sum_{i\in S_{j}}u_{i}\left(S_{j},\pi \right)$, where $u_{i}\left(S_{j},\pi \right)$ is the individual payoff of UAV-i in coalition $S_{j}\in \pi$.*

(17) $$\begin{array}{c} u_{i}\left(S_{j},\pi \right)=\sum_{t\in T_{S_{j}}}R_{i}\left(t\right) \end{array}$$

*$R_{i}\left(t\right)$ is the throughput at time slot t of UAV-i, which is illustrated in (8), and $T_{S_{j}}$ is the available time slots of the coalition $S_{j}\in \pi$. Since each UAV-i has a fixed transmitting time slot i, $T_{S_{j}}=S_{j}=\left\{j_{1},j_{2},\ldots ,j_{\left|S_{j}\right|}\right\}$. In this sense, the disjoint coalition can also be seen as the coalition of time slots.*

Note that $v\left(\pi \right)$ can also be described as the system payoff, which is calculated as the sum of the payoff of each coalition in π. This coalition game has a non-transferable utility (NTU), which means that the payoff of each UAV in a coalition $S_{j}$ cannot be arbitrarily allotted.

It is not hard to understand that the utility function satisfies the hyper-additive $\forall S_{i},S_{j}\in \pi$; if $S_{i}\cap S_{j}=Ø$, then $v\left({S_{i}\cup S}_{j}\right)\geq v\left(S_{i}\right)+v\left(S_{j}\right)$.

As an example, in Figure 1, we assume that the 16 UAVs in the multi-UAV network form a given partition: $\pi =\left\{\left\{1\right\},\left\{2\right\},\left\{3,16\right\},\right.\left.\left\{4,8,13\right\},\left\{5,6,7\right\},\left\{9,10,11\right\},\left\{12,14,15\right\}\right\}$. The payoff of UAV-1 is $u_{1}\left(\left\{1\right\},\pi \right)=R_{0}$, since there is only one player in the coalition and one slot to transmit. Then, the utility of the coalition $\left\{1\right\}\;$ is $v\left(\left\{1\right\},\pi \right)=R_{0}$. In the coalition $\left\{3,16\right\}$, the two elements are so far away that they have no collision with each other. Thus, UAV-3 and UAV-16 can transmit packets in the two time slots $\left\{3,16\right\}$ simultaneously, the payoff of UAV-3 or UAV-16 is $u_{3}\left(\left\{3,16\right\},\pi \right)=u_{16}\left\{\left\{3,16\right\},\pi \right\}={2R}_{0}$, and the utility of coalition $\left\{3,16\right\}$ is $v\left(\left\{3,16\right\},\pi \right)=u_{3}\left(\left\{3,16\right\},\pi \right)+{u_{16}\left\{\left\{3,16\right\},\pi \right\}=4R}_{0}$. While in coalition $\left\{5,6,7\right\}$, the arbitrary element is in the communication range of another element, the time slots $\left\{5,6,7\right\}$ cannot be shared to transmit packets simultaneously, and the payoff of an arbitrary UAV in the coalition is $u_{i}\left(\left\{5,6,7\right\},\pi \right)=R_{0}$. Therefore, the utility of coalition $\left\{5,6,7\right\}$ is $v\left(\left\{5,6,7\right\},\pi \right)=3R_{0}$. The utility of the remaining coalitions can be calculated in the same manner. Thus, the utility of the coalitional structure π can be calculated as $v\left(\pi \right)=\sum_{j=1}^{8}v\left(S_{j},\pi \right)=25$.

The solution to the time slot coalition game $G_{D}=\left(S,\;V\right)$ is to find a stable coalitional structure. Moreover, the newly stable coalitional structure should have the properties of external stability and internal stability.

In the coalition formation game, the action of each UAV is to form a coalition $S_{i}$, where *i* is the index of the coalition. Given the current coalition structure, i.e., the state of the game, the UAV faces three options: (i) stay in the current coalition, (ii) join with any of the other coalitions, or (iii) leave the current coalition. Here, we define the join-and-leave rule.

**Definition** **4** (Join-and-leave rule)**.** *In the time slot coalition game $G_{D}=\left(S,\;V\right)$ given the coalitional structure $\pi_{x}$, two coalitions $S_{j},S_{k}\in \pi_{x}$, j≠k and node $i\in S_{k}$. If $\pi_{x}{≻}_{i}\pi_{y}$, node i can decide to leave coalition $S_{k}$ and join coalition $S_{j}$, and $\pi_{x}$ can evolve to $\pi_{y}$: $\pi_{y}=\left\{\pi_{x}\setminus \left(S_{k},S_{j}\right)\right\}\cup \left\{S_{k}\setminus i\right\}\cup \left\{S_{j}\cup \left\{i\right\}\right\}$. That is, $\pi_{x}{≻}_{i}\pi_{y}$ is represented as $\pi_{x}{≻}_{i}\pi_{y}\Leftrightarrow u_{i}\left(\left\{S_{j}\cup \left\{i\right\}\right\},\pi_{y}\right)>u_{i}\left(S_{k},\pi_{x}\right)$,$v\left(\left\{S_{j}\cup \left\{i\right\}\right\},\pi_{y}\right)>v\left(S_{j},\pi_{y}\right)\;$ and $\;v\left(\pi_{y}\right)>v\left(\pi_{x}\right)$. During time slot coalition formation, each UAV-i prefers to join another new coalition and leave its current coalition if its individual payoff, the new coalitional payoff, and the system payoff increase.*

**Definition** **5** (The stable partition)**.** *For a given partition $\pi^{*}=\left(S_{1},S_{2},\ldots ,S_{M}\right)$, if $\forall i\in S_{k}$, $\forall S_{j}\in \pi^{*},j\neq k$, $u_{i}\left(S_{k},\pi^{*}\right)\geq u_{i}\left(\left\{S_{j}\cup \left\{i\right\}\right\},\pi^{\prime }\right)$, $v\left(S_{k},\pi^{*}\right)\geq v\left(\left\{S_{k}\setminus i\right\},\pi^{\prime }\right)$ and $v\left(\pi^{*}\right)\geq v\left(\pi^{\prime }\right)$, where $\pi^{\prime }=\left\{\pi^{*}\setminus \left(S_{k},S_{j}\right)\right\}\cup \left\{S_{k}\setminus i\right\}\cup \left\{S_{j}\cup \left\{i\right\}\right\}$, then $\pi^{*}$ is a stable partition. In a stable partition, there does not exist a UAV that wants to leave the current coalition to join another one.*

### 4.2. Distributed Coalition Formation Algorithm

Next, we propose a distributed time slot coalition formation algorithm based on the join-and-leave rule. The algorithm has three phases: discovery of near UAVs, adaptive coalition formation, and joint transmission. Algorithm 1 summarizes the distributed coalition formation algorithm.

Phase 1: Each UAV discovers neighboring UAVs in the range of $d_{C}+d_{I}$ in order to share the time slots in the coalition for no collision transmission. The network discovery and information collection can be based on a fixed control time slot allocated to every UAV.

Phase 2: Following the discovery phase, adaptive coalition formation begins, and UAVs mutually intend to form a time slot coalition. Then, an iteration of sequential join-and-leave runs depending on the utility improvement. The adaptive coalition formation terminates until there is no any UAV obtaining utility improvement between two iterations.

Phase 3: Once a coalition structure is formed, the UAVs in a coalition start transmitting packets at the sharing time slots simultaneously.
**Algorithm 1** Procedure of Distributed Time Slot Coalition Formation Algorithm 1   **Phase 1:** Discovery of neighbors 2   **Phase 2:** Adaptive coalition formation 3     **Initialization:** t=0 and $\pi \left(t\right)=\left\{S_{1}\left(t\right),\ldots ,S_{M}\left(t\right)\right\}$ 4     **Repeat** 5        (1) For each UAV-*i* calculates the $u_{i}\left(S_{k}\left(t\right),\pi \left(t\right)\right)$, $v\left(S_{k}\left(t\right),\pi \left(t\right)\right)$             and $v\left(\pi \left(t\right)\right)$, where $i\epsilon S_{k}\left(t\right)$; 6        (2) UAV-*i* randomly chooses a coalition $S_{q}\left(t\right),i\notin S_{q}\left(t\right)$ to join,             calculates $u_{i}\left(\left\{S_{q}\left(t\right)\cup \left\{i\right\}\right\},\pi^{\prime }\left(t\right)\right)$, $v\left(\left\{S_{q}\left(t\right)\cup \left\{i\right\}\right\},\pi^{\prime }\left(t\right)\right)$             and $v\left(\pi^{\prime }\left(t\right)\right)$,
where $\pi^{\prime }\left(t\right)$ denotes the coalition structure             after UAV-*i* joining $S_{q}\left(t\right)$; 7        (3) **If** $\pi \left(t\right){≻}_{i}\pi^{\prime }\left(t\right)$, **then**:             UAV-*i* decides to join the coalition $S_{q}\left(t\right)$;             $\pi^{\prime }\left(t\right)=\left\{\pi \left(t\right)\setminus S_{k}\left(t\right),S_{q}\left(t\right)\right\}\cup \left\{S_{k}\left(t\right)\setminus i\right\}\cup \left\{S_{q}\left(t\right)\cup \left\{i\right\}\right\}$;             $\pi \left(t+1\right)$ can be replaced to $\pi^{\prime }\left(t\right)$; 8           **End if**; 9        (4) t=t+1;  10     **Until** Convergence to a stable coalition structure.  11   **Phase 3:** The UAVs in a coalition start transmitting packets at the sharing   time slots simultaneously.

### 4.3. Performance Analysis

**Propriety** **1.** *The coalition formation process based on the join-and-leave rule converges to a final stable partition in a finite number of steps.*

**Proof.**  When the join-and-leave rule operates, it may transform the current coalition partition into another partition. Consequently, there exists a sequence of switch operations, i.e., $\pi^{\left(0\right)}\to \pi^{\left(1\right)}\to \pi^{\left(2\right)}\to \cdots$. As the number of players, i.e., UAVs, is finite in our proposed game, the coalitions they can form are also finite, and the number of coalition structures is also finite. Therefore, from any $\pi^{\left(0\right)}$, the sequence always terminates to a stable partition $\pi^{*}$ after finite iterations. Furthermore, the joining and leaving of coalitions can happen only when it leads to utility improvement in the new coalition and coalition structure; no loop appears during the process, meaning that the number of steps is bounded. Since the coalition structure changes only when the utility is increased and there must be a maximum utility for the limited number of coalition structures, the coalition formation process must converge to a final stable partition in a finite number of steps. If $\pi^{*}$ is not stable, there exists a UAV that can perform the join-and-leave operation. According to the Pareto order, an arbitrary UAV switches from the current coalition to another only when its utility increases without hurting the utilities of other coalition members in both the original and new coalition. Then, the arbitrary UAV does switch its coalition to pursue higher utility. Hence, $\pi^{*}$ is not the final stable partition, which contradicts the previous argument. Consequently, the final state must be stable. Therefore, Propriety 1 is proven.  □

**Propriety** **2.** *The complexity order of the proposed Algorithm 1 is* $O\left(N{*D}_{N}\right)$*.*

**Proof.**  The complexity of Algorithm 1 lies in the complexity of the join-and-leave operations. For a given multi-UAV network, there are $D_{N}$ different coalition structures for *N* UAVs. In this algorithm, during each time step *t*, every UAV compares its current individual payoff with the corresponding payoff after it joins to a new coalition. Then, the total number of comparisons required to converge to a final stable partition is $O\left(N*D_{N}\right)$, as the maximum comparison number for every UAV is $D_{N}-1$. However, the complexity of the algorithm can be significantly reduced by leveraging the historical information set, preventing nodes from joining or leaving the same coalition more than once.  □

## 5. Designs of TDMA Protocol-Based Distributed Coalition Formation Games

**Figure 3:**
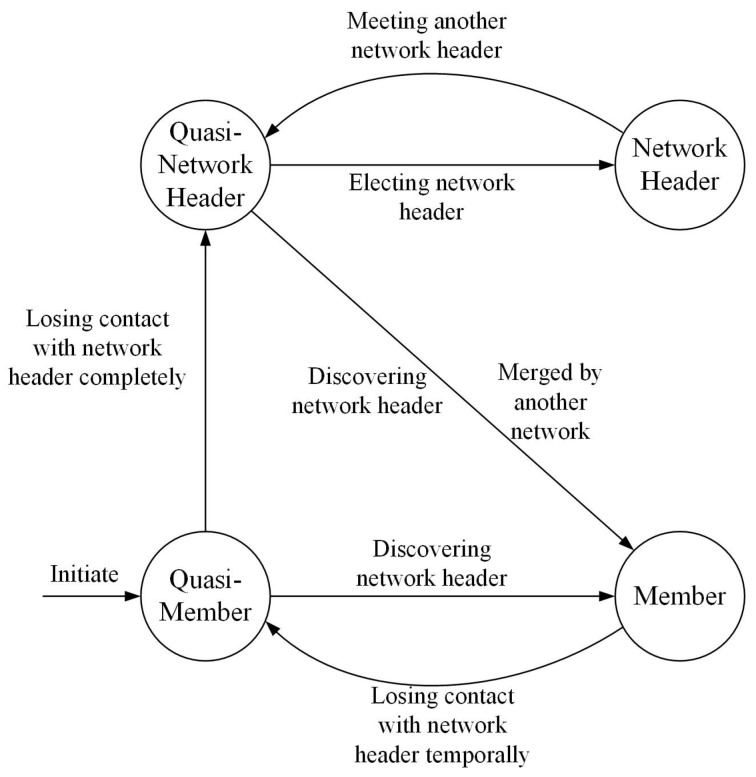
FSM of our proposed scheme.

At a given time, each UAV operates under only one state with a different function. As shown in Figure 3, a finite-state machine (FSM) is employed to precisely describe the principle and the operating process of our proposed scheme. In the distributed coalition formation (DCF) slot allocation scheme, the UAVs go through four different states: (1) network header; (2) quasi-network header; (3) member; (4) quasi-member.

In the first state, NH (network header), the UAV node is the organizer of the DCF game for the whole network, which starts the DCF game and confirms the coalition structure. The second state, quasi-network header, indicates that the UAV node is neither a network header nor a member. When a member node completely loses contact with the network header, it enters the QNH (quasi-network header) state. In the third state, the member state, the UAV node executes the join-and-leave rule and sends a request to the network header. In the last state, the quasi-member state, the UAV node tries to find the signal of the network header.

### 5.1. Network Configuration Protocol

The state transitions of the FSM depicted in Figure 3 are controlled by the Network Configuration Protocol. The state-transition conditions are described as follows:

(1) Initiation: When a UAV node initially starts up, it enters the quasi-member state.
(2) Discovering network header: When the quasi-member and quasi-network header UAV node receive the invite-to-join (ITJ) message from the network header again, they transform to the member state.
(3) Losing contact with the network header temporally: When the member UAV node cannot receive the schedule assignment broadcast periodically from the network header over the limit time, the state of the UAV node changes from member to quasi-member.
(4) Losing contact with network header completely: When the quasi-member UAV node cannot receive the schedule assignment ITJ message two consecutive times, the UAV node enters the quasi-network header state. The network header will delete this UAV node from the member list if it cannot receive packets from this UAV node three consecutive times.
(5) Electing network header: When acting as a quasi-network header over a limited time, this UAV node elects itself as a network header and broadcasts the ITJ advertisement message periodically. The other quasi-network header UAV node joins its network and forwards the ITJ advertisement message when it receives the ITJ advertisement message.
(6) Meeting another network header: When a network header receives another valid ITJ advertisement message, it enters the quasi-network header state and compares the node identification with another network header. The UAV network header with smaller identification then becomes a new network header.
(7) Merging by another network: When a network header receives another valid ITJ advertisement message, the UAV network header with smaller identification merges another network. The UAV network header with larger identification joins the new network and changes to the member state.

### 5.2. Time Frame Structure

**Figure 4:**
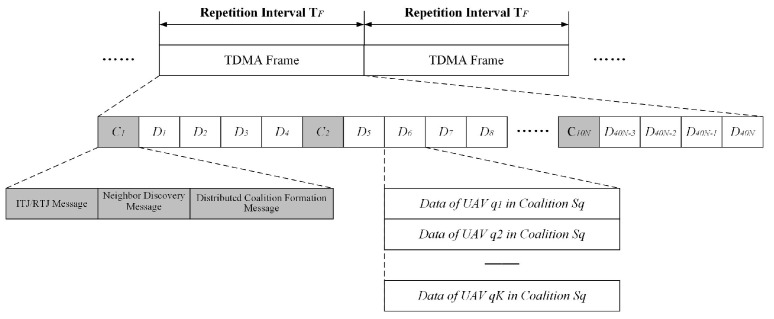
Time frame structure.

In the multi-UAV network, time is partitioned into regular time intervals with equal lengths of $T_{F}$, which is called the repetition period. As shown in Figure 4, the repetition period consists of two kinds of time slots: (1) control slots and (2) data slots. The repetition interval is divided into 50 N equal-length slots. The length of each time slot that is assigned to each member, which is denoted by $t_{slot}$, can be determined by $t_{slot}=T_{F}/N$.

There are 10 N control slots and 40 N data slots in the repetition interval $T_{F}$. The control slot, which is denoted by $C_{i}$, includes the ITJ/RTJ message, neighbor discovery message and distributed coalition formation message. In the data slot $D_{i}$, the UAV nodes included in the same coalition can transmit data simultaneously. Obviously, in this design, control slots occupy effective data transmission time, which decreases the performance of network throughput. In Figure 4, there are 10 N control slots in one repetition interval, which consists of 50 N slots. The overhead of the control slots is 20%.

### 5.3. Neighbor Discovery Protocol

The neighbor discovery protocol is designed to obtain the neighbor list within a three-hop range. Every UAV node broadcasts a neighbor discovery message (NDM) to its one-hop neighbors in their pre-allocated control slots. The neighbor discovery message includes its neighbor list within a two-hop range. UAV nodes exchange their two-hop neighbor lists after sending and receiving NDMs. Then, the nodes which are included in the neighbor’s NDM can be increased to the neighbor list within a three-hop range. In the DCF slot allocation scheme, the join-and-leave rule is operated based on the neighbor list within a three-hop range. We make an approximation assumption that the three-hop range is longer than $d_{C}+d_{I}$, and the neighbors out of the three-hop range can share the same sending slot of the center UAV node.

### 5.4. Slot Allocation Protocol of DCF Game

In the slot allocation protocol, UAV nodes are divided into one network header and the members. They transmit data at pre-allocated slots after time synchronization. We assume that UAV nodes in the multi-UAV network undergo time synchronization via a GPS system. As shown in Figure 4, the control time slot $C_{ik}$ is pre-allocated to UAV-*i*, *k* = 1:10, and the data time slot $D_{ik}$ is pre-allocated to UAV-*i*, *k* = 1:40. Before the slot allocation protocol operates, the UAV nodes send control and data packets in their own pre-allocation slots. Each node in the network achieves its three-hop neighbors via the neighbor discovery protocol, which runs periodically at the control slots.

The network header is the organizer of the DCF game for the whole network, which starts the DCF game and confirms the coalition structure. The member UAV node operates the join-and-leave rule and sends a request-of-new-coalition (ROC) message to the network header. The network header collects all ROC messages of the whole member UAV nodes and decides the next coalition structure. All the messages are transmitted to the pre-allocated control slot. The detailed slot allocation process is described as follows:

(1) The network header broadcasts the DCF message to the whole network in its control slot. The DCF message includes the current coalition structure $\pi \left(t\right)=\left\{S_{1}\left(t\right),\ldots ,S_{M}\left(t\right)\right\}$ with a sequence number, which is confirmed by the network header.
(2) Every member node forwards the DCF message to other nodes once in the form of a broadcast at the pre-allocated control slot after it receives and stores the coalition structure $\pi \left(t\right)$. All UAV nodes transmit data packets in the data slots based on the slot allocation scheme defined by the coalition structure in the next repetition interval.
(3) An ROC message is generated after operating the join-and-leave rule based on the coalition structure of the DCF message broadcast by the network header. Then, the member node sends the ROC message to the network header in the form of a broadcast.
(4) Other member nodes forward the ROC message once in the form of a broadcast to ensure that it can be received by the network header.
(5) After every member node has sent ROC messages, the network header decides the new coalition message structure based on the arrival order of ROC messages. Arriving earlier means the node is closer to the network header and its pre-allocated slot is in the front.
(6) The network header broadcasts the new DCF message, which includes the new coalition structure, to the whole network.
(7) Step 1 to step 6 are repeated in the whole network running time. The data packets are transmitted to the data slots allocated by the coalition structure simultaneously without waiting for the Algorithm 2 to converge.

**Algorithm 2** Procedure of DCFG-TDMA protocol1UAV-*i* goes online;2Wait for the broadcast signaling ITJ of the network header;3**If** receive the ITJ broadcast signal from network header, **then:**4    Join the network, enter in the member state, and get the current coalition partition;5    Operate Algorithm 1, participate in the iterative process of DCFG-TDMA slot allocation;6    Transmit data on the allocated time slot;7**Else:**8    Continue waiting and enter the associate member status;9    **If** receving a ITJ in two repetition intervals, **then:**10      Join the network, enter the member state;11      Repeat 2–6;12    **Else**, donot receive a ITJ in 3 repetition intervals, **then:**13      Enter quasi-network header state;14      Broadcast ITJ signal;15      **If** receive RTJ from other UAV nodes,**then:**16         Enter the network header state, broadcast ITJ signal periodically;17         Run Algorithm 1, maintain the time slot allocation process of the network;18      **Else if** receive a ITJ from other network header, **then:**19         Compare the node id of itself and other network header;20         **If** my id smaller than another ITJ node, **then:**21           I am the network header, continue broadcasting ITJ signal;22         **Else:**23           Join another network, and become a member;24           Repeat 2–6.

## 6. Numerical Results

In this paper, we use as an example a square area of $50\times 50{\mathrm{km}}^{2}$ in the suburban environment, where *S* UAV nodes are uniformly distributed. The simulation parameters are shown in Table 2. All UAV nodes operate at a carrier frequency of 2.4 GHz with an available bandwidth of 20 MHz. The data rates of UAV nodes for control packets and data packets are R=20 Mbps. In reference [38], the value of the attenuation factors $\eta_{los}$ is 1. Considering the difference in noise floor between suburban and urban environments, the variance of noise $\sigma^{2}$ is set to $5\times 10^{-19}$ W/Hz. The communication range can be calculated, and is 2225.4 m. Based on the above parameters, we simulate the static grid and dynamic topology cases using MATLAB R2018a.

### 6.1. Static Grid Topology Environment

**Figure 5:**
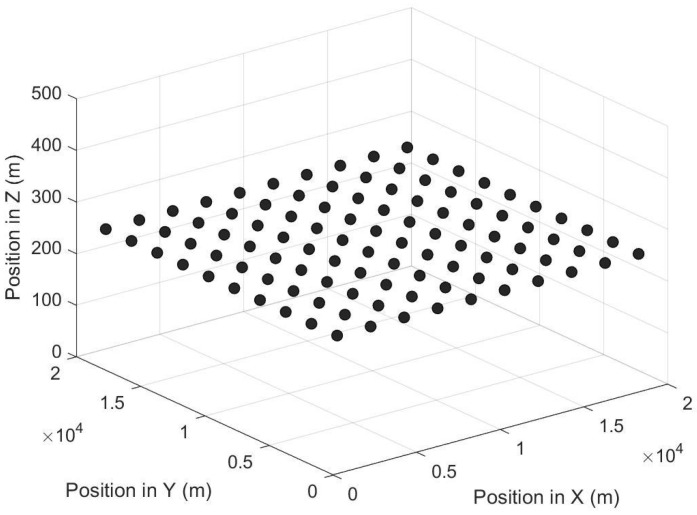
Grid topology involving 100 UAV nodes.

We consider a static grid topology involving 100 UAV nodes. Figure 5 shows the 100-UAV-node grid topology, where the distance between two neighboring UAVs is 2 km.

**Figure 6:**
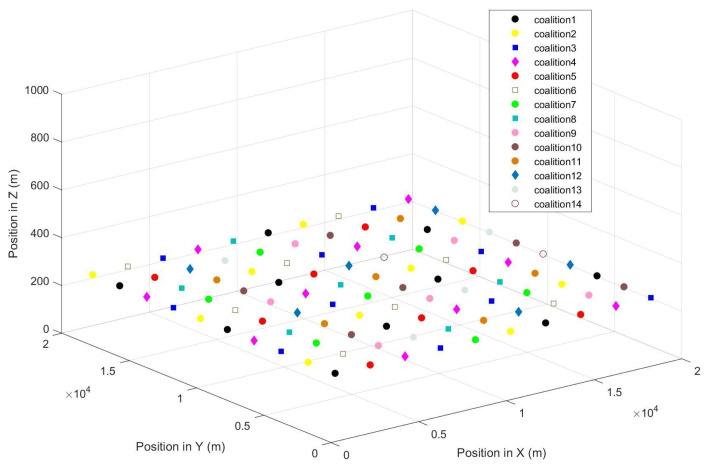
The final coalition structure for the 100-UAV-node grid topology.

**Figure 7:**
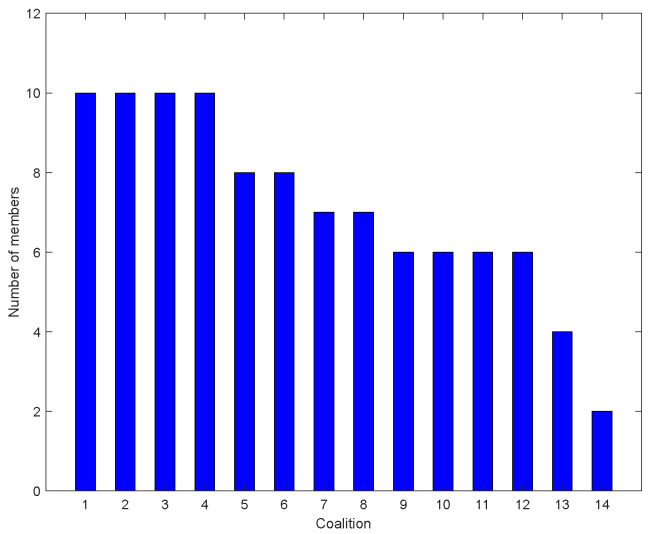
The number of members in each coalition.

Figure 6 shows the coalition structure once it has finally converged. In the end, the network is divided into 14 coalitions. The maximum number of coalition members is 10. Figure 7 gives the member number of each coalition. We find that the number of coalition members decreases with the coalition sequence, because the UAV nodes in the front join the coalition first. The UAV nodes in coalition 1 achieve 10 transmitting slots in a repetition interval.

**Figure 8:**
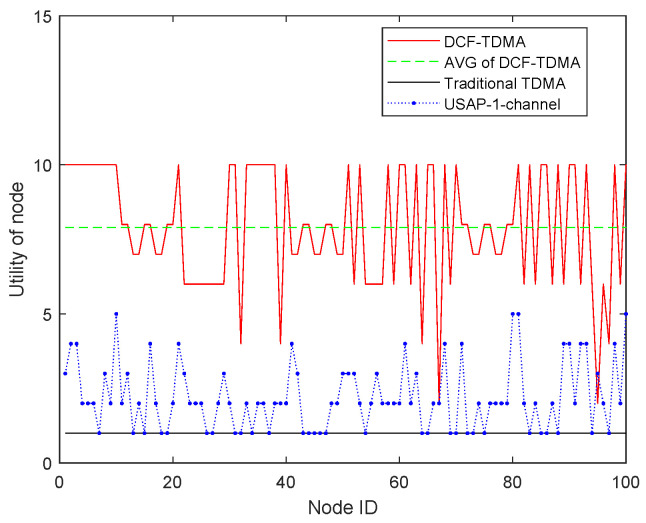
The utilities of every UAV node.

The utilities of every UAV node are given in Figure 8. The average utility of DCFG-TDMA proposed in this paper is 7.9 slots. The max utility and the min utility of DCF-TDMA are 10 and 3 slots, respectively. Compared to that of the Traditional TDMA and USAP, the average utility is improved significantly. This is because, although the USAP algorithm is capable of reusing time slot resources, the distributed selection process still encounters numerous conflicts, making it impossible to achieve an optimal time slot allocation outcome.

**Figure 9:**
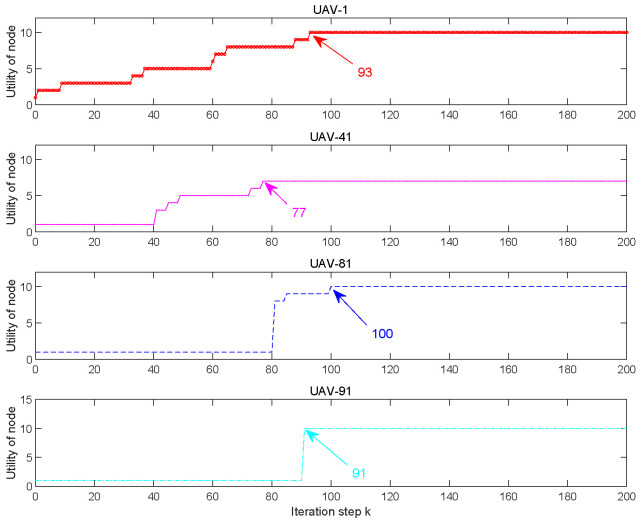
The utilities vs. iteration steps.

The utilities of four UAV nodes (UAV-1, UAV-41, UAV-81, UAV-91) versus the iteration step are shown in Figure 9. UAV-1 and UAV-91 achieve the maximum utility at step 93, UAV-41 achieves the maximum utility at step 77, and UAV-81 achieve the max utility at step 100.

**Figure 10:**
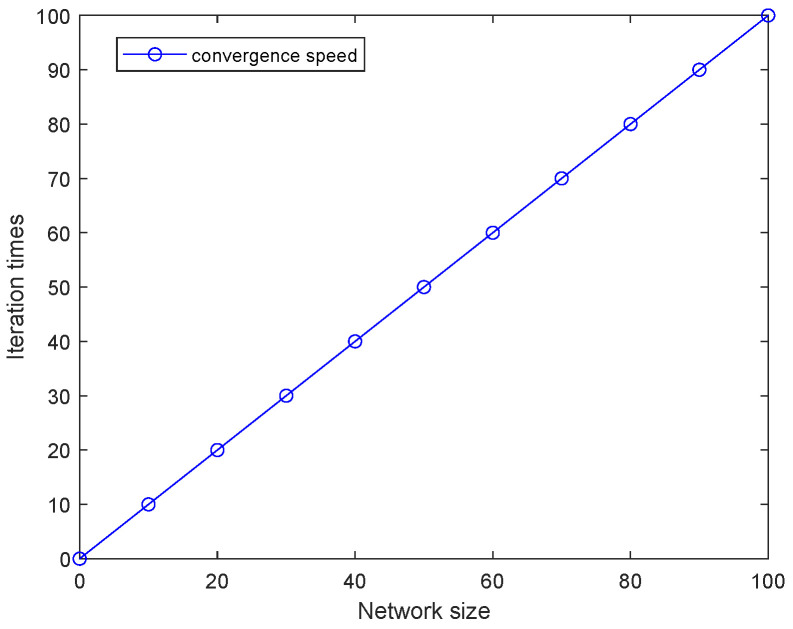
Convergence speed vs. network size.

The convergence speed of the algorithm versus network size is given in Figure 10. The number of iterations is linearly related to the network size for the grid topology, which is a regular topology in which each UAV node achieves stable partitioning within one iteration.

### 6.2. Dynamic Topology Environment

**Figure 11:**
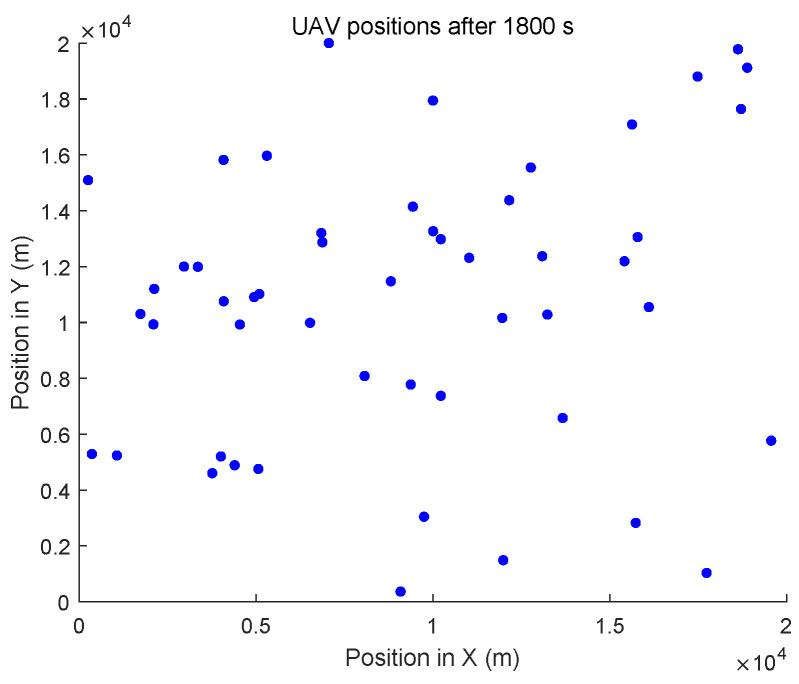
UAV positions after 1800 s.

In the dynamic topology environment, UAVs randomly select a direction and a speed (≤5 m/s) for their flight. The initial positions of all UAVs are different. The positions after flying for 1800 s are shown in Figure 11.

**Figure 12:**
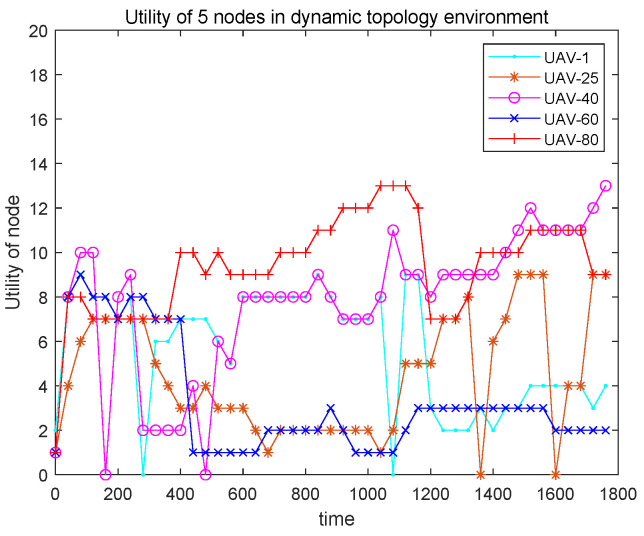
The utilities of the 5 UAV nodes in a dynamic topology environment.

Figure 12 shows the utilities of five UAV nodes versus simulation time. With the proposed DCF-TDMA protocol, UAV nodes can achieve time slots adaptively with a rapid change in topology. For UAV-1, its utility arrives at the first high point, which is 9 at the 80th s. At the 480th s, UAV-1 achieves the minimal time slots, because the topology changes significantly such that the coalition formatted before cannot obtain any benefit. Therefore, the protocol automatically operates the join-and-leave mechanism. UAV-1 quickly achieves many correct transmission slots again in a few seconds. UAV-1 achieves 11 transmission slots between the 760th s and 1120th s. The maximum number of transmission slots for UAV-1 is 17. From the curve of UAV-1, we can see that the topology changes significantly at the 480th, 640th, 1160th, and 1480th s. Compared to those of UAV-1 and UAV-80, the transmission slot number of UAV-40 is much smoother.

**Figure 13:**
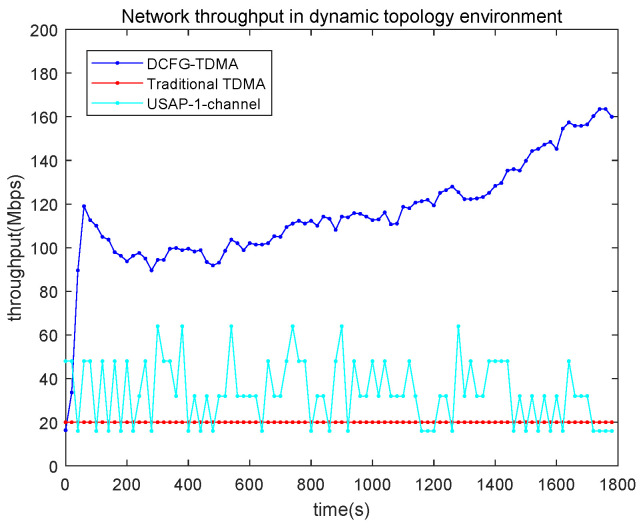
Network throughput in a dynamic topology environment.

The performance of network throughput in the dynamic topology environment is shown in Figure 13. Compared to traditional TDMA and USAP, the throughput of the proposed DCFG-TDMA protocol improves significantly.

## 7. Conclusions

In this paper, we study the internal interference problem in UAV swarm ad hoc networks, where the performance of UAVs is severely affected by interference. A distributed slot allocation algorithm for UAV swarm ad hoc networks based on the time coalition formation game is proposed. In this method, the UAV nodes with no interference with each other autonomously form a coalition to achieve the greatest benefit. UAV nodes in the same time coalition can share their time slots and transmit data packets simultaneously. In this context, the DCFG-TDMA protocol is designed for UAV swarm ad hoc networks with dynamically changing network topology to adaptively form time coalitions. Our simulation results verified that the proposed DCFG-TDMA can successfully allocate time slots in a UAV swarm ad hoc network and provide significant gains in terms of the average utility per UAV node and network. 

## Figures and Tables

**Table 1:** Key variables used in this paper.

Variables	Explanation
*M*	The number of coalitions
*N*	The number of UAVs and slots
N	The index set of the UAVs
T	The set of the available time slot
*B*	The bandwidth of a channel
$R_{0}$	The fixed data rate
$P_{t}$	The transmit power of each UAV
$d_{C}$	The communication range
$d_{I}$	The interference range
$P_{r}$	The received power of the signal
$G_{C}=\left\{V,E_{C}\right\}$	The communication graph
$G_{I}=\left\{V,E_{I}\right\}$	The collision graph
$G_{D}$	A distributed coalition game with non-transferable utility
S	The finite set of players (i.e., UAVs)
V	A partition function
$\prod \left(S\right)$	A partition of S
$u_{i}\left(S_{j},\pi \right)$	The individual payoff of UAV i in coalition $S_{j}\in \pi$
$v\left(S_{j},\pi \right)$	The utility function of the coalition $S_{j}\in \pi$
$v\left(\pi \right)$	The system payoff on π
$T_{n}$	The corresponding slots selected by UAV-*n*
Th	The throughput of the whole multi-UAV network

**Table 2:** Simulation parameter settings.

Parameter	Value
Carrier frequency *f*	2.4 GHz
Bandwidth *B*	20 MHz
Channel parameter $\eta_{los}$	1
Variance of noise $\sigma^{2}$	$5\times 10^{-19}$ W/Hz
UAV transmit power $p_{t}$	0.5 W
Data rate *R*	20 Mbps
Communication range $d_{C}$	2225.4 m
Slot	100 μs
Repetition interval	500 ms

## Data Availability

The data presented in this study are available on request from the corresponding author.

## References

[1] Letaief K.B., Shi Y., Lu J., Lu J. (2022). Edge rrtificial intelligence for 6G: Vision, enabling technologies, and applications. IEEE J. Sel. Areas Commun..

[2] Zeng Y., Zhang R., Lim T.J. (2016). Wireless communications with unmanned aerial vehicles: Opportunities and challenges. IEEE Commun. Mag..

[3] Mozaffari M., Saad W., Bennis M., Nam Y.-H., Debbah M. (2019). A tutorial on UAVs for wireless networks: Applications, challenges, and open problems. IEEE Commun. Surv. Tutorials.

[4] Khan M.A., Kumar N., Mohsan S.A.H., Khan W.U., Nasralla M.M., Alsharif M.H. (2023). Swarm of UAVs for network management in 6G: A technical review. IEEE Trans. Netw. Serv. Manag..

[5] Qi F., Zhu X., Mang G., Kadoch M., Li W. (2019). UAV Network and IoT in the Sky for Future Smart Cities. IEEE Netw..

[6] Chen J., Chen P., Wu Q., Xu Y., Qi N., Fang T. (2021). A Game-theoretic Perspective on Resource Management for Large-scale UAV Communication Networks. China Commun..

[7] Ding G., Wu Q., Zhang L., Lin Y., Tsiftsis T.A., Yao Y.-D. (2018). An Amateur Drone Surveillance System Based on the Cognitive Internet of Things. IEEE Commun. Mag..

[8] Zhao J., Gao F., Wu Q., Jin S., Wu Y., Jia W. (2018). Beam Tracking for UAV Mounted SatCom on-the-Move with Massive Antenna Array. IEEE J. Sel. Areas Commun..

[9] Zeng Y., Wu Q., Zhang R. (2019). Accessing From the Sky: A Tutorial on UAV Communications for 5G and Beyond. Proc. IEEE.

[10] Wu B., Guo D., Zhang B., Zhang X., Wang H., Wang H., Jiang H. (2022). Completion Time Minimization for UAV Enabled Data Collection with Communication Link Constrained. IET Commun..

[11] Wang H., Wang J., Ding G., Chen J., Gao F., Han Z. (2019). Completion Time Minimization with Path Planning for Fixed-wing UAV Communications. IEEE Trans. Wirel. Commun..

[12] Wu B., Zhang B., Ma W., Xie C., Guo D., Jiang H. (2023). Motion Planning in UAV-Aided Data Collection with Dynamic Jamming. Electronics.

[13] Wu B., Zhang B., Guo D., Wang H., Jiang H. (2022). Anti-jamming trajectory design for UAV-enabled wireless sensor networks using communication flight corridor. China Commun..

[14] Gao Y., Wu Y., Cui Z., Yang W., Hu G., Xu S. (2022). Robust Trajectory and Communication Design for Angle-constrained Multi-UAV Communications in the Presence of Jammers. China Commun..

[15] Samir M., Sharafeddine S., Assi C.M., Nguyen T.M., Ghrayeb A. (2020). UAV Trajectory Planning for Data Collection from Time-Constrained IoT Devices. IEEE Trans. Wirel. Commun..

[16] Fei B.L.Z., Zhang Y. (2019). UAV Communications for 5G and Beyond: Recent Advances and Future Trends. IEEE Internet Things J..

[17] Dorling K., Heinrichs J., Messier G.G., Magierowski S. (2017). Vehicle Routing Problems for Drone Delivery. IEEE Trans. Syst. Man Cybern. Syst..

[18] Zhan C., Zeng Y. (2019). Completion Time Minimization for Multi-UAV-Enabled Data Collection. IEEE Trans. Wirel. Commun..

[19] Borgonovo F., Capone A., Cesana M., Fratta L. (2004). ADHOC MAC: New MAC Architecture for Ad Hoc Networks Providing Efficient and Reliable Point-to-point and Broadcast Services. Wirel. Netw..

[20] Omar H.A., Zhuang W., Li L. (2013). VeMAC: A TDMA-based MAC protocol for reliable broadcast in VANETs. IEEE Trans. Mob. Comput..

[21] Nguyen V., Dang D.N.M., Jang S., Hong C.S. E-VeMAC: An Enhanced Vehicular MAC Protocol to Mitigate the Exposed Terminal Problem. Proceedings of the 16th Asia-Pacific Network Operations and Management Symposium (APNOMS 16).

[22] Zou R., Liu Z., Zhang L., Kamil M. A near collision free reservation based MAC protocol for VANETs. Proceedings of the IEEE Wireless Communications and Networking Conference (WCNC).

[23] Jiang A., Mi Z., Dong C., Wang H. CF-MAC: A Collision-free MAC Protocol for UAVs Ad-Hoc Networks. Proceedings of the IEEE Wireless Communications and Networking Conference (WCNC).

[24] Chua M.Y.-K., Yu F.R., Li J., Zhou Y., Lamont L. (2013). Medium Access Control for Unmanned Aerial Vehicle (UAV) Ad-Hoc Networks With Full-Duplex Radios and Multipacket Reception Capability. IEEE Trans. Veh. Technol..

[25] Wang H., Jiang B., Zhao H., Zhang J., Zhou L., Ma D. (2022). Joint Resource Allocation on Slot, Space and Power Towards Concurrent Transmissions in UAV Ad Hoc Networks. IEEE Trans. Wirel. Commun..

[26] Xu Y., Anpalagan A., Wu Q., Shen L., Gao Z., Wang J. (2013). Decision-Theoretic Distributed Channel Selection for Opportunistic Spectrum Access: Strategies, Challenges and Solutions. IEEE Commun. Surv. Tutorials.

[27] Ahmed M., Peng M., Abana M., Yan S., Wang C. (2018). Interference Coordination in Heterogeneous Small-Cell Networks: A Coalition Formation Game Approach. IEEE Syst. J..

[28] Wang B., Wang L., Fu G., Liu W., Cui J. A New Distributed Coalition Formation Algorithm for Cooperation in Ad Hoc Networks. Proceedings of the 2018 IEEE 4th International Conference on Computer and Communications (ICCC).

[29] Li T., Li C., Yang C., Shao J., Zhang Y., Pang L. (2021). A Mean Field Game-theoretic Cross-layer Optimization for Multi-hop Swarm UAV Communications. J. Commun. Netw..

[30] Zhang Z., Atapattu S., Wang Y., Renzo M.D. (2025). Distributed MAC for RIS-Assisted Multiuser Networks: CSMA/CA Protocol Design and Statistical Optimization. IEEE Trans. Mob. Comput..

[31] Peng G., Fang D., Fu B., Zhang N. NCSMA: A NOMA-Based CSMA/CA Protocol for Ad Hoc Networks. Proceedings of the 2024 IEEE 35th International Symposium on Personal, Indoor and Mobile Radio Communications (PIMRC 24).

[32] Young C.D. USAP multiple access: Dynamic resource allocation for mobile multihop multichannel wireless networking. Proceedings of the IEEE Military Communications 1999.

[33] Zhang S., Ni Z., Kuang L., Jiang C., Zhao X. (2022). Load-Aware Distributed Resource Allocation for MF-TDMA Ad Hoc Networks: A Multi-Agent DRL Approach. IEEE Trans. Netw. Sci. Eng..

[34] Zhao X., Wei Z., Zou Y., Ma H., Cui Y., Feng Z. (2024). A dual-cluster-head based medium access control for large-scale UAV ad-hoc networks. China Commun..

[35] Sohaib M., Jeong J., Jeon S.-W. (2022). Dynamic Multichannel Access via Multi-agent Reinforcement Learning: Throughput and Fairness Guarantees. IEEE Trans. Wirel. Commun..

[36] Liu X., Xu C., Yu H., Zeng P. (2022). Deep Reinforcement Learning-Based Multichannel Access for Industrial Wireless Networks With Dynamic Multiuser Priority. IEEE Trans. Ind. Inform..

[37] Naeem F., Adam N., Kaddoum G., Waqar O. Learning MAC Protocols in HetNets: A Cooperative Multi-Agent Deep Reinforcement Learning Approach. Proceedings of the 2024 IEEE Wireless Communications and Networking Conference (WCNC).

[38] Li X., Yao H., Wang J., Xu X., Jiang C., Hanzo L. (2019). A Near-Optimal UAV-Aided Radio Coverage Strategy for Dense Urban Areas. IEEE Trans. Veh. Technol..

